# Evaluating Functional Immunity Following Encapsulated Bacterial Infection and Vaccination

**DOI:** 10.3390/vaccines9060677

**Published:** 2021-06-20

**Authors:** Zheng Quan Toh, Rachel A. Higgins, Nadia Mazarakis, Elysia Abbott, Jordan Nathanielsz, Anne Balloch, Kim Mulholland, Paul V. Licciardi

**Affiliations:** 1 Murdoch Children’s Research Institute, Parkville, VIC 3052, Australia; zheng.quantoh@mcri.edu.au (Z.Q.T.); Rachel.higgins@mcri.edu.au (R.A.H.); Nadia.mazarakis@mcri.edu.au (N.M.); elysiaabbott@outlook.com (E.A.); jmnathanielsz@gmail.com (J.N.); anne.balloch@mcri.edu.au (A.B.); kim.mulholland@lshtm.ac.uk (K.M.); 2 Department of Paediatrics, The University of Melbourne, Parkville, VIC 3052, Australia; 3 London School of Hygiene and Tropical Medicine, University of London, London WC1E 7HT, UK

**Keywords:** functional antibodies, assays, opsonophagocytosis, serum bactericidal assays, encapsulated bacteria

## Abstract

Encapsulated bacteria such as *Streptococcus pneumoniae*, *Haemophilus influenzae* type b and *Neisseria meningitidis* cause significant morbidity and mortality in young children despite the availability of vaccines. Highly specific antibodies are the primary mechanism of protection against invasive disease. Robust and standardised assays that measure functional antibodies are also necessary for vaccine evaluation and allow for the accurate comparison of data between clinical studies. This mini review describes the current state of functional antibody assays and their importance in measuring protective immunity.

## 1. Introduction

Invasive bacterial infections caused by encapsulated bacteria such as the *Streptococcus pneumoniae* (pneumococcus), *Haemophilus influenzae* type b (Hib) and *Neisseria meningitidis* (meningococcus) are the leading causes of morbidity and mortality in children under five years of age globally [1,2]. Together, they are responsible for more than half a million deaths each year, with most of these cases occurring in low- and middle-income countries (LMICs) [1,2]. The high burden of disease in LMICs is largely attributed to limited access to vaccines and appropriate healthcare [2].

The pneumococcus, Hib and meningococcus are commensal bacteria that colonise the human upper respiratory tract [3]. Colonisation by these bacteria is necessary to cause invasive bacterial diseases. In healthy individuals, colonisation is usually asymptomatic and does not lead to disease, but in certain populations, such as children <5 years of age or adults >65 years of age, and those who are immunocompromised (i.e., HIV-infected, asplenic patients and those who have undergone solid organ transplant), colonisation by these bacteria can cause serious diseases such as pneumonia, meningitis and sepsis [4].

Highly specific antibodies generated by the host immune response are the primary mechanism of protection against bacterial colonisation and disease [5], although cellular immune responses such as Th17 and regulatory T cells are also thought to be involved in preventing bacterial colonisation [6,7]. These antibodies bind to the capsular polysaccharides surface of the bacterium, effectively blocking infection, and can also act as opsonins that elicit bacterial clearance by recruiting immune factors (complement) and innate immune cells (neutrophils or macrophages) [8]. Functional antibodies that mediate bacterial clearance are an important measure of protective immunity.

Functional antibody assays such as serum bactericidal assays (SBA) and opsonophagocytic assays (OPA) are used to measure antibody-mediated clearance of encapsulated bacteria. Currently, different methods have been used to evaluate functional antibodies, making comparison of immunogenicity data from different studies difficult, particularly in clinical trial settings. Robust and standardised assays are critical for licensure of new vaccines and for evaluating vaccine immunogenicity, including alternate vaccination schedules such as reduced doses or extended intervals between doses [9,10,11]. Alternate vaccine schedules that are more cost-effective and logistically friendly are particularly relevant for LMICs and remote settings. Many LMICs have limited laboratory capacity and may have difficulty performing functional antibody assays, particularly the OPA. Nevertheless, a standardised assay that is feasible to implement would be of significant value. Such assays could be transferred from an established laboratory of another country to build capacity, or alternatively there are also World Health Organization (WHO) reference laboratories that can provide technical support for countries wishing to establish these assays.

## 2. Functional Antibody Assays against Encapsulated Bacteria

**Figure 1:**
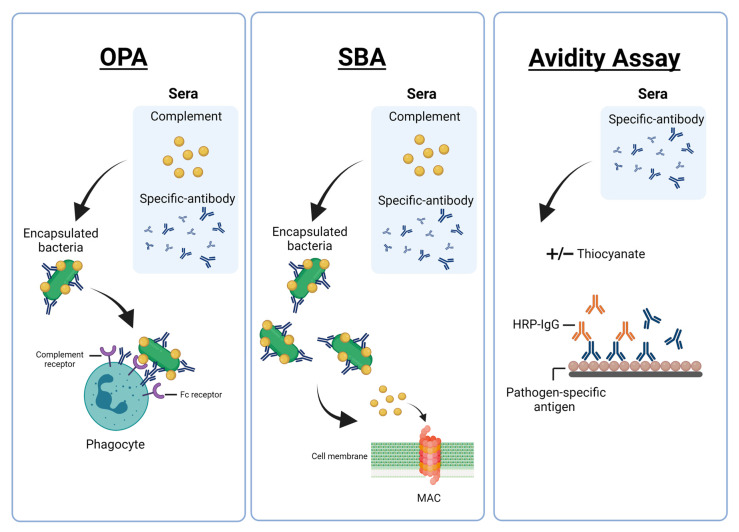
Theoretical concept of opsonophagocytic assays (OPA), serum bactericidal assay (SBA) and avidity assay. OPA: antigen-specific antibodies along with complement proteins opsonise encapsulated bacteria and facilitate uptake of the antibody-bacteria complex by phagocytes. SBA: antigen-specific antibodies recruit complement proteins that activate the complement cascade. This leads to the formation of the membrane attack complex (MAC) in the bacterial cell membrane, resulting in bacterial cell lysis. Avidity assay measures the strength of the antigen-antibody binding and is usually performed using a modified enzyme-linked immunosorbent assay (ELISA). Chaotropic agents such as thiocyanate are incubated with serum to elute antibodies that bind weakly to the antigen.

Encapsulated bacteria are mainly cleared via a type-specific antibody through complement-mediated killing and/or opsonophagocytosis. The functional capacity of the antibodies can be measured by assays such as OPA, SBA and antibody avidity assays. The theoretical concepts of OPA, SBA and antibody avidity assays are described in Figure 1, and their advantages and disadvantages summarised in Table 1.

Recently, there has been growing interest in evaluating the Fc-mediated function of the antibody response in addition to the antibody binding activity facilitated by the Fab region [12]. ‘Systems serology’ is a relatively new approach that uses data-driven computational analysis and high-throughput experimental data to interrogate important antibody features associated with protective humoral immunity and/or Fc functional activity. The Fc region of the antibody is important in activating a range of antibody-dependent (AD) immune responses such as AD cellular cytotoxicity, AD cellular phagocytosis, AD complement activity and AD cytokine, chemokine and enzyme release [12]. While some of these mechanisms are the basis for OPA and SBA, these antibody-dependent activities may act independently or in combination to control bacterial growth and survival. Using systems serology may therefore aid in elucidating novel functional antibody mechanisms associated with pneumococcal, Hib and meningococcal bacteria, although more research is needed.

## 3. Importance of Measuring Functional Antibodies Following Vaccination

Measurement of functional immunity is a critical aspect of vaccine evaluation and is often required by regulators for new vaccine licensure [13]. The absolute correlates of protection for pneumococcal, Hib and meningococcal opsonophagocytic or bactericidal antibodies are summarised in Table 2 [5].

Functional antibody assays except antibody avidity assay require in vitro or ex vivo cell and bacterial cultures, which are labour intensive and time consuming to perform. As a result, ELISA and electrochemiluminescence-based immunoassay [14] are more commonly used to measure antibody concentrations to define protection and are more easily able to set up in LMICs. Generally, higher antibody concentration indicates better protection. However, this is not always the case since the ELISA method measures functioning and non-functioning antibodies, including those that are not involved in protection.

The level of functional antibodies against these encapsulated bacteria following immunisation appears to be dependent on the population, serotype, and the clinical endpoint (disease or carriage) assessed. In certain age groups and demographics, such as the very young and elderly adults or immunocompromised individuals, as well as in countries with a high burden of disease, discordance in antibody levels and functions following immunisation have been reported for pneumococcus [15,16,17], Hib [18,19,20] and meningococcus [21,22]. This discordance is likely due to the generation of non-functioning or weakly binding antibodies, including reduced antibody diversity, defects in isotype switching, and/or a lack of somatic mutation [20,23]. Deficiency in IgM memory B cells is also more commonly seen in the elderly [24]. There is also the possibility of ‘antigen sin’ where previous exposure to cross-reactive antigens may induce non-functional antibodies in the case for Hib [20,25]. Discordant pneumococcal antibody levels and function for certain pneumococcal serotypes, particularly Serotype 1, have also been reported following immunisation with 10- and 13-valent pneumococcal conjugate vaccines [26,27]. Therefore, some of the correlates of protection need to be better defined, especially by standardised functional assays.

## 4. Standardisation of Functional Antibody Assays against Encapsulated Bacteria

Assay standardisation is crucial for the reliability of data and for the comparison of study findings in vaccine evaluation and surveillance studies. Earlier versions of OPA or SBA methods differed in reagents, including the bacterial strains, complement source and effector cells (for OPA, donor neutrophil vs. HL-60). These parameters can influence the outcome measurements, making it challenging to compare results across different laboratories and studies. This is also true for antibody avidity assays where there is also a need for standardisation [28].

Standardisation of functional assays has been successful for pneumococcal bacteria and, to a certain extent, for Meningococcal A and C (Table 3). Selection of target strains in the SBA assay is of critical importance for evaluating meningococcal antibodies. Meningococcal strains for capsular groups A and C have been recommended for use in a standardised SBA assay [29]; however, there is currently no consensus on strains for Groups B, W135 and Y. Although there has been effort to standardise Meningococcal B (MenB) SBA [30], no formal method has been established. One of the challenges for standardising SBA against Serogroup B is the diverse epidemiology of prevalent strains in different populations [31,32], making the choice of a ‘universal’ reference strain difficult. In addition, selection of a bacterial strain that is susceptible (i.e., the alternative and lectin activation pathways, which do not require an antibody) or resistant (i.e., the production of bacterial proteins that interfere with complement killing and/or the overproduction of bacterial polysaccharide) to complement killing may not be suitable for use in the assay [33]. There have been efforts to evaluate and validate SBA against Hib [34], but no standardised protocol has been established and no standardisation exercise among laboratories has been conducted. A new high throughput SBA to measure functional antibodies to Hib has recently been developed using frozen bacteria and the automated colony counting method based on agar plates with a chromogenic dye [35]. This assay was found to correlate strongly with anti-Hib IgG antibody levels, although further assay standardisations are required.

The complement proteins from intrinsic human serum or exogenous sources such as human or animal sera (i.e., baby rabbit complement) used in SBA can have a profound effect on study results [36]. While human serum is the preferred complement source for SBA when evaluating human immunity, it usually contains endogenous antibodies that may interfere with the assay. Baby rabbit complement was found to be a suitable source of complement for SBA to minimise variability, although it generally results in comparatively higher titres when compared to using human complement [36].

To enable comparison across different studies, a standardised assay is preferred by regulators, such as the pneumococcal OPAs. This may serve as a reference for the establishment of OPA for other bacterial pathogens, particularly those that involve antibody clearance through complement-killing as well as opsonophagocytosis.

## 5. OPA for Evaluating (New) Bacterial Vaccines

The killing OPA for pneumococcus has been successful in evaluating pneumococcal functional antibodies. The use of standardised reagents minimises assay variability and enables comparison of results from different clinical studies. Both Hib and meningococcal bacteria are susceptible to complement-dependent serum bactericidal and opsonising antibodies [37]. Immunisation with meningococcal polysaccharide vaccines was found to elicit both complement-dependent serum bactericidal and opsonising antibodies [38,39]. The development of standardised OPAs for meningococcus would be a worthwhile exercise, but there have been very few developments in this space.

Two different versions of meningococcal OPA have been described previously: a polysaccharide-specific flow cytometric-based OPA using HL-60 cells [40], and a method measuring respiratory burst activity using donor polymorphonuclear neutrophils [41]. Both were found to strongly correlate with SBA. More recently, an OPA has been developed for meningococcal B using the immune dominant outer membrane proteins rather than the capsular polysaccharides. The use of OPA to evaluate MenB vaccine immunity has been particularly important in patients with primary terminal complement deficiency. Since SBA relies exclusively on complement-mediated killing, studies in children and adults with primary terminal complement deficiencies found no serum bactericidal activity against MenB. However, complement-dependent opsonophagocytic killing was still detectable in these individuals, suggesting that they might still be protected following vaccination [38,42]. Measurement of this immune mechanism in this immunocompromised cohort may also be relevant to vaccines against other bacterial pathogens. We have developed a Hib OPA based on the pneumococcal OPA method and found the method to be a more sensitive assay for assessing functional Hib vaccine-induced immunity compared to SBA, and the results reproducible (P Licciardi, personal communication). This represents a novel way to evaluate Hib antibodies, although further testing and validation in larger cohorts are needed. There has also been the development of OPA for non-encapsulated bacteria such as Group A Streptococcus (GAS) and Group B Streptococcus (GBS) to evaluate candidate vaccines [43,44,45]. Both methods were adapted from the pneumococcal OPA method using HL-60 cells and baby rabbit complement which eliminate major sources of variations seen with the use of donor neutrophils and complement.

## 6. Conclusions

Functional antibody assays are important for the measurement of protective antibodies against encapsulated bacteria. They are often labour intensive and time consuming, and current study methods vary considerably between laboratories. Standardisation of assay parameters eliminates major sources of assay variations, enabling the comparison of data from population studies and/or vaccination trials more accurately. The pneumococcal OPA method has been standardised and allows for multiplexing. Recently, the development of OPA for other non-encapsulated bacteria such as GAS and GBS has been adapted from the pneumococcal OPA method. Future development of OPA for other bacteria will be of interest, particularly for other encapsulated bacteria that are susceptible to both antibody-mediated complement killing and opsonophagocytosis.

## Figures and Tables

**Table 1:** Advantages and disadvantages of currently available functional assays.

Assays	Advantages	Disadvantages
Traditional Killing OPA/MOPA pneumococcal-specific	Standardised gold-standard assay	Labour intensive Time consuming Can have high repeat rate ^
Fluorescent OPA/MOPA pneumococcal-specific	Single-day assay Eliminates colony-counting Semi-automation	Non-standardised output Requires specialised equipment (i.e., flow cytometer or fluorometer) Variable results for some serotypes
Serum Bactericidal Assay Hib and meningococcal	Does not require phagocytic cell line	Non-standardised reagents Does not measure opsonophagocytic activity Time consuming
Antibody Avidity Assay pneumococcal, Hib and meningococcal	Easy to perform Does not require live bacteria	Non-biological assay Non-standardised method (dilution vs. elution)

^ due to a difference in antibody levels for different serotypes within each MOPA panel.

**Table 2:** Correlates of protection for pneumococcal, Hib and meningococcal vaccines.

Vaccines	Correlates of Protection
PCV	ELISA >0.35 µg/mL OPA ≥8 titre
Hib	ELISA Long term: ≥1.0 µg/mL Short term: >0.15 µg/mL SBA ≥4 titre
Meningococcal *	SBA rSBA (≥8 titre) or hSBA (≥4 titre)

Data obtained from [5]. PCV: Pneumococcal conjugate vaccine. Hib: *Haemophilus influenzae* type b. ELISA: enzyme-linked immunosorbent assay. OPA: opsonophagocytic assay. SBA: serum bactericidal assay. rSBA: rabbit complement serum bactericidal assay. hSBA: human complement serum bactericidal assay. * only been formally correlated with effectiveness for Serogroup C; no defined cut-off for ELISA as correlate of protection.

**Table 3:** Standardised OPA and SBA for evaluating pneumococcal and meningococcal ACYW vaccines.

Bacterial	Assay	Bacterial Strains	Complement Source	Immune Cells	Reference
Pneumococcal	OPA	Serotype-specific strains available through BEI resources	Baby Rabbit complement	HL-60	[11]
Meningococcal A and C	SBA	Serogroup A strain F8238 Serogroup C strain C11	Baby Rabbit complement	NA	[29]

NA: not applicable.
